# Mother or Father: Who Is in the Front Line? Mechanisms Underlying the Non-Genomic Transmission of Obesity/Diabetes via the Maternal or the Paternal Line

**DOI:** 10.3390/nu11020233

**Published:** 2019-01-22

**Authors:** Bernard Portha, Valérie Grandjean, Jamileh Movassat

**Affiliations:** 1 Sorbonne-Paris-Cité, Laboratoire B2PE (Biologie et Pathologie du Pancréas Endocrine), Unité BFA (Biologie Fonctionnelle et Adaptative), Université Paris-Diderot, CNRS UMR 8251, F-75205 Paris CEDEX 13, France; bernard.portha@univ-paris-diderot.fr; 2 Inserm U1065 C3M, Team Control of Gene Expression (10), Université Côte d’Azur, 151 Route de Ginestière, 06204 Nice CEDEX 3, France; Valerie.Grandjean@unice.fr

**Keywords:** maternal and paternal metabolic imprinting, germ cell epigenome, sncRNAs, DOHaD, obesity, diabetes

## Abstract

Extensive epidemiological and experimental evidence have shown that exposure to an adverse intrauterine environment as observed in offspring of pregnancies complicated by obesity or diabetes, can program susceptibility to metabolic, endocrine and cardiovascular disorders later in life. Although most studies have concentrated on the maternal environment, it is also becoming evident that paternal exposure to obesity or diabetes can result in the later development of metabolic disorders in the offspring. Such programmed effects might not be limited to the first directly exposed generation, but could be transmitted to subsequent generations. This suggests the existence of mechanisms by which metabolic changes in parental phenotype are transmissible to offspring. The mechanisms which underpin the transmission of the programmed effects across generations are still unclear. However, epigenetic regulation of transcription has emerged as a strong candidate for mediating the heritability of metabolic diseases. Here, we review the most relevant evidence from human and animal studies showing transmission of programming effects of obesity or diabetes across generations, and the current mechanisms underlying either maternal or paternal influences on the metabolic status of offspring.

The prevalence of obesity and type 2 diabetes (T2D) worldwide has reached pandemic proportions. This is believed to be the result of complex interactions between an individual’s genome and environmental cues. Although single nucleotide polymorphisms at multiple genetic loci have been shown to be associated with T2D, for the majority of people with T2D, only a small proportion (5% to 10%) can be explained by genetic background [1]. Environmental factors such as consumption of a high-fat, high-sugar diet and/or inactivity and their interaction with the genome, are thought to be critical in the determination of T2D risk [2,3].

The Developmental Origins of Health and Disease (DOHaD) concept has highlighted the crucial importance of the fetal and early postnatal environment in shaping long-term health [4], and a substantial body of evidence links parental nutritional status to metabolic traits in offspring. Extensive epidemiological and experimental evidence has shown that exposure to an adverse intrauterine environment as observed in the offspring of pregnancies complicated by obesity or diabetes can program susceptibility to metabolic, endocrine and cardiovascular disorders in later life [5]. Although most studies have concentrated on the maternal environment, it is also becoming evident that paternal exposure to obesity or diabetes can result in the later development of metabolic disorders in the offspring [5]. Such programmed effects might not be limited to the first directly exposed generation, but could be transmitted to subsequent generations. This suggests mechanisms by which nutritionally induced changes in parental phenotype are transmissible to offspring [6]. The mechanisms which underpin the transmission of the programmed effects across generations are still unclear. However, epigenetic regulation of transcription has emerged as a strong candidate for mediating the heritability of metabolic diseases [7]. Here, we review the most relevant evidences from human and animal studies showing transmission of programming effects of obesity or diabetes across generations, and the mechanisms underlying either maternal or paternal influence on the metabolic status of offspring. For the sake of clarity, we focused our review on the more abundant studies using rodent models. One must not forget however that many experimental studies have been conducted in larger species, such as sheep, pigs and non-human primates.

## 1. Obesity or Diabetes Risk Can Be Transmitted across Generations via the Maternal Line. Epidemiological and Experimental Evidence

Obesity is a major risk factor for type 2 diabetes (T2D) development [8]. Recent studies have demonstrated that not only does obesity in adulthood increase the risk of developing T2D, but obesity during pregnancy can increase the risk of diabetes in the offspring through non-genetic mechanisms. This is of major concern because the current global obesity epidemic includes women of reproductive age. In addition to the immediate detrimental consequences of maternal overweight/obesity during pregnancy for the mother, there is now strong evidence for long-term detrimental consequences for offspring metabolic health. There are now many cohorts ongoing to estimate the association between mother and progeny’s BMI. While for some of them, the impact of mother BMI on metabolic health of offspring might be better explained by genetic and lifestyle factors than a causal epigenetic inheritance [9,10,11,12], few of them indicate that obesity and/or diabetes risk might be transmitted across generations via the presence of epigenetic modification in the maternal germ line as it will be discussed below.

Thus, studies of siblings born before or after bariatric surgery to reduce maternal weight, have shown that those born prior to surgery have increased adiposity, increased blood pressure and reduced insulin sensitivity compared to those born after maternal surgery [13]. On the other side, retrospective studies of human cohorts exposed to poor nutrition during prenatal and early postnatal life, provided evidence to support similar detrimental consequences for offspring. Studies of the Dutch “hunger winter”, a period of abrupt onset of severe famine during German blockade of the Netherlands at the end of the Second World War, have been informative. Poor nutrition experienced by pregnant mothers during the famine was associated with increased fat mass, hypertension, glucose tolerance, and psychiatric disorders emerging in their children during adult life [14,15]. Similar findings have been demonstrated for populations around the world. The Ukraine famine (1932–1933) also revealed associations between early gestational nutrient restriction and development of T2D in adulthood [16]. Similarly, individuals born immediately after periods of famine in Austria (1918–1919, 1938, 1946–1947) [17] or China (1959–1961) have higher rates of T2D or hyperglycemia [18]. Scarcity of food during the Biafran famine during the Nigerian Civil War (1967–1970) and resulting undernutrition during gestation and childhood have also been associated with impaired glucose tolerance in adulthood [19]. Therefore, both low birth weight (especially when followed by accelerated postnatal growth) and high birth weight is associated with increased risk of obesity, T2D and other features of the metabolic syndrome in childhood, adolescence and adult life [20]. This fuels an intergenerational cycle of obesity and T2D that is independent of the genotype.

The role of maternal T2D inheritance has also been recognized in a majority of epidemiological studies [21,22]. Early evidence for this has been seen in the offspring of Pima Indian women with pre-existent T2D or gestational diabetes. These offspring were larger for gestational age at birth and were heavier and had impaired glucose tolerance (IGT) in childhood compared to offspring of pre-diabetic or non-diabetic women [23]. These findings indicate that intrauterine exposure to a diabetic environment increases the risk of obesity and T2D beyond that attributable to genetic factors, at least in Pima Indians. To circumvent the confounding effects of genes linked to early-onset T2D and transmitted by the pregnant T2D mother, the effect of fetal exposure to type 1 diabetes (T1D) was evaluated in adult offspring lacking T1D immunological markers. A 33% prevalence of impaired glucose tolerance was reported in the offspring of T1D mothers compared with none in those with T1D fathers (control group) [24,25]. Altogether, these findings suggest that fetal exposure to maternal diabetes is indeed associated with abnormal glucose homoeostasis in offspring and may be involved in maternal T2D transmission. In adult Pima Indians with normal glucose tolerance and whose mothers get T2D, insulin secretion in response to glucose was reduced in those offspring whose mothers had been diabetic before pregnancy, whereas it remained normal in those whose mothers had developed diabetes after pregnancy [25]. Body fat and insulin sensitivity were similar in the two groups of offspring [25]. The offspring of T1D mothers also had reduced insulin secretion, but similar fat mass and insulin activity compared with the offspring of T1D fathers [24]. Again, in the non-diabetic offspring of mothers with young-onset T2D (diagnosed at age <50 years), insulin secretion was decreased compared with the offspring of fathers with young-onset T2D [26]. Thus, human studies suggest that a defect of insulin secretion is involved in the abnormal glucose tolerance observed in adult offspring exposed to maternal diabetes. More importantly, they found that insulin secretion might be reduced even in normal glucose-tolerant offspring. The classical studies by Hales et al. [27], evidenced a several-fold increase in the incidence of glucose intolerance and T2D in adult men who were born small compared with those born with normal birth weight. This pattern was correlated to inadequate beta cell secretion [28]. The fetal period is indeed critical for endocrine pancreatic development in rodents and in humans [24], and the clinical data show that children and adults with low birth weights have impaired beta cell function compared with their normal-birth-weight counterparts [29,30]. Indeed, one report concluded that human fetuses with severe growth retardation have a reduction in pancreatic endocrine cell mass on autopsy [31]. However, as it is not possible to measure beta cell mass in vivo, this hypothesis cannot be further tested directly in humans.

Experimental studies support epidemiological observations and have provided strong evidence for transmission of the obese and diabetic phenotype from parent to offspring through non-genetic mechanisms. Numerous studies in rodents have investigated the effects of maternal obesity obtained in response to high-fat (HF) only, or high-fat/high-sugar diet, before and/or throughout pregnancy and during lactation [32]. Overnutrition and obesity in the F0 dam can also yield phenotypes in F2 and F3 generations [33,34]. Despite the differences in diet composition, and length of maternal overnutrition, most of the studies showed increased offspring adiposity, insulin resistance, and finally development of poor glucose tolerance and T2D, which has been attributed to a combination of beta cell dysfunction [35] and insulin resistance [36,37,38]. One must not forget that abnormalities in beta cell function are critical in defining the T2D risk, because T2D installs only when beta-cell function deteriorates and fails to compensate for insulin resistance in peripheral tissues [8]. Prenatal and/or early postnatal exposure to undernutrition also causes increased adiposity and glucose intolerance/diabetes in the offspring (F1) [39,40] and reduction of the number and function of pancreatic islets [41]. It also increased adiposity and glucose intolerance in the next (F2) generation [42,43]. Moreover, if an undernutrition insult is sustained, there can be further propagation of metabolic phenotypes across many generations. When Wistar rats were subjected to 50% caloric restriction over 50 generations, offspring had fasting hyperinsulinemia, glucose intolerance, and increased adiposity. The impaired metabolic phenotype was not reversed by restoration of nutrition for two generations [44]. In rat models of spontaneous diabetes, early beta cell alterations with decreased beta cell mass have been reported in fetuses from both spontaneously diabetic BB rats (T1D model) [45] and spontaneously diabetic GK rats (T2D model) [46]. On evaluating the long-term consequences for the progeny in these models, IGT was observed in the offspring of mildly streptozotocin (STZ)-induced diabetic females due to lower insulin secretion in response to glucose, while insulin resistance was reported in the offspring of severely STZ-diabetic mothers [47,48,49]. Glucose tolerance was also impaired in the offspring of normal mothers receiving glucose infusions during late gestation, and was associated with decreased glucose-induced insulin secretion [50]. Since most of these models of diabetes in pregnancy have drawbacks (see discussion in [51]), we have proposed that embryo transfer experiments might represent a more relevant paradigm [52]. When fertilized Wistar rat oocytes were transferred into diabetic GK female rats and the neonates were suckled by non-diabetic Wistar foster mothers, beta cell mass in the F1 offspring was decreased at fetal and adult ages, and impaired glucose tolerance was present at adult age (review in [51]). Control rats originating from Wistar oocyte transfer to normal Wistar females retained normal glucose tolerance. Therefore, maternal spontaneous diabetes shapes offspring beta cell mass and insulin secretion. Such a scenario is relevant to the GK rat model of spontaneous T2D [53] since the GK mothers are mildly hyperglycemic through their gestation and during the suckling period. This could represent one mechanism for initiation of pancreas programming in the F1 offspring of the first founders (F0), since the GK line is issued from intercrosses between females and males Wistar with borderline IGT but otherwise normal basal blood glucose level [53,54]. This could also contribute to the lack of attenuation of the diabetic GK phenotype over time [53,54].

## 2. Critical Windows of Exposure to Maternal Obesity/Diabetes Shape Metabolic Risk in the Offspring

**Figure 1:**
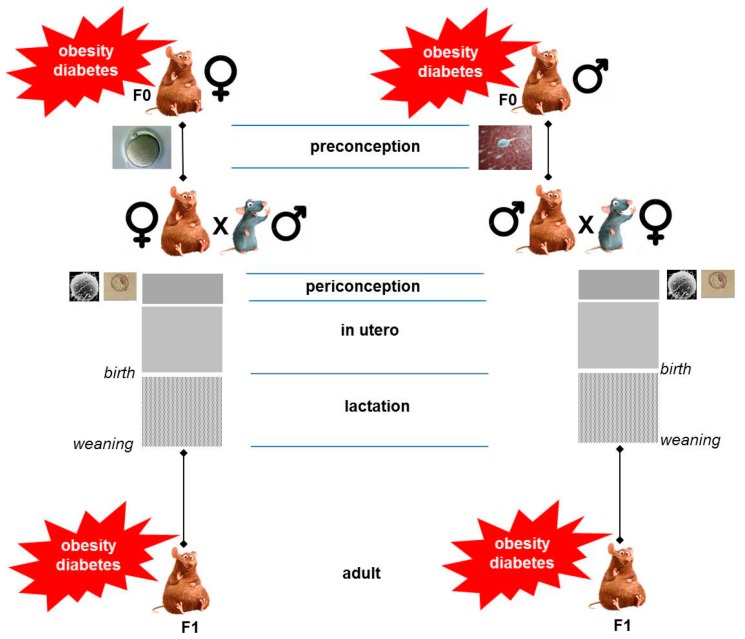
Parental contribution for programming adult obesity/diabetes in the offspring. The preconception (oocyte maturation, follicular development), conception (fertilization and embryo growth until implantation), gestational and suckling periods are critical time-windows for maternal obesity/diabetes (brown rodent) to shape the risk for the offspring to develop obesity or diabetes (left panel). The preconception period (sperm cell maturation) is a critical period for paternal obesity/diabetes (brown rodent) to shape the risk for the offspring to develop obesity or diabetes (right panel).

Since it is now well described that obesity or diabetes in pregnancy induces multiple phenotypic consequences in offspring, it is important to recognize the windows of exposure to the maternal insult that are responsible for conferring these effects (Figure 1).

Both fetal and suckling periods have been shown to be critical time-windows in determining long-term metabolic heath. A large number of animal studies employing early weaning, cross-fostering, or nutritional manipulation during the lactation period only [55] support the notion that the early postnatal period is critical for insulin resistance associated with obesity, or insulin deficiency associated with diabetes, to develop in the offspring. It is also admitted that exposure to an adverse metabolic milieu prior to pregnancy accounts for some of the adverse outcomes observed in offspring. Previous studies suggest that obesity/diabetes have adverse effects on the oocyte and embryo [56]. Using reciprocal mouse embryo transfers, one can experimentally achieve the separation of a pre-gestational exposure to a given maternal insult, from the effects of an *in utero* exposure. When applied to the impact of maternal obesity (HF diet) [57], it was found that a pre-gestational exposure to maternal obesity impaired fetal and placental growth with a marked alteration of the expression of imprinted genes and genes regulating vasculogenesis and lipid metabolism in the placenta (despite a normal gestational milieu) [57]. An exposure to maternal obesity only during gestation also resulted in fetal growth restriction and decreased placental weight. However, gestational exposure to maternal obesity resulted in obesity and impaired glucose tolerance only in the adult offspring, suggesting that their obese phenotype is not related to placental dysfunction [57].

## 3. Maternal Obesity or Diabetes Remodels Offspring Epigenome

Epigenomic information established during early development plays widespread roles in maintaining cell phenotypes across the mitotic generations throughout the lifetime of the organism. Some of this information (marks) has a potential to be influenced by environmental factors (diet, physical activity, chemical exposure, stress) and to get disrupted by being erased, reestablished or modified. Accordingly, they represent the best candidates underlying persistent effects of the fetal environment, later in life after termination of the challenge. It is known that the metabolic state of the organism directly influences epigenetic modifications, as DNA/histone methylation/acetylation rely upon substrates derived from intermediary metabolism such as S-adenosyl methionine, acetyl CoA, α-ketoglutarate, and nicotinamide adenine dinucleotide [58]. For example, abnormal metabolic and mental health were reported in adult offspring whose mothers were exposed to the Dutch Hunger Winter during the peri-conceptional period [14], and these changes were associated with DNA methylation changes in key growth and metabolic genes in whole blood samples of the adult offspring [59,60]. Similarly, the methylome changes correlated with changes in gene expression level and they were associated with reduced obesity and improved metabolic risk profile reported in siblings born after maternal gastrointestinal bypass surgery compared with siblings born before maternal surgery [13]. Abnormal maternal metabolic health during pregnancy such as gestational diabetes or obesity in human or rodents, has also been shown to be associated with epigenetic changes in metabolic genes [61]. Gestational obesity has been associated with altered expression levels of circulating microRNAs such as miR-340, miR-423-5p, and miR-652 [62]. These differences in microRNA expression levels correlated with changes in placental weight and birth weight [58]. Moreover, changes in expression levels of miR-29c, miR-128a, and miR-221 correlated with body weight at six months of life [62]. Taken together, these studies suggest that epigenetic modifications could serve as a potential biomarker of the later-life offspring metabolic health in at-risk population. However, these human studies should be interpreted with caution, since the significance of the modest DNA methylation differences (less than 10%) observed in mixed cell populations is questionable, and extrapolation from epigenetic changes encountered in cord blood, placenta, or whole blood to non-accessible target tissues may be erroneous, as epigenetic changes are highly specific to tissues and cell types.

A number of animal models have been used to assess the role of epigenetics in pathogenesis of obesity and diabetes in the offspring [63,64]. Epigenetic modifications affecting the expression of key genes critical for endocrine pancreas development and beta cell function, peripheral glucose uptake, and insulin action have been identified in offspring of various models of metabolic abnormalities during pregnancy including diabetes and obesity, and obtained in various species (rodents, sheep, guinea pigs, and non-human primates). They have been correlated with the profound changes in offspring pancreas, liver, skeletal muscle, and adipose tissue, and have been proposed as one mechanism that could contribute to the development of later-life metabolic disorders (see detailed review in [65]). One of the most comprehensive models in this perspective is the utero-placental insufficiency (UPI) model generated in female rat via uterine bilateral artery ligation. UPI offspring develop late T2D in adulthood [66]. Epigenetic modifications playing a critical role in the expression of key genes that contribute to the diabetic phenotype, have been described in detail in this model [67]. Both genome-wide and locus-specific epigenetic changes have been studied in UPI offspring target tissues. For example, by performing genome-wide methylation studies, 1400 differentially methylated loci in isolated islets from adult male UPI offspring were found [68]. A majority of the differentially methylated CpG sites were found at conserved intergenic regions and near genes that had been previously described as regulators of beta cell division and death, vascularization, and insulin secretion [68]. Among the locus-specific changes in islets of UPI offspring, the expression of the pancreatic homeobox domain 1 (*Pdx1*) gene was permanently reduced in beta cells and this contributed significantly to the diabetic phenotype reported in this model [67]. *Pdx1* is a key developmental transcription factor gene which regulates early pancreatic development and later differentiation and function of the beta cells [69]. This reduced *Pdx1* expression was epigenetically modulated by binding of mSin3A-HDAC complex to the *Pdx1* promoter leading to histone deacetylation, followed by a reduction in H3K4 trimethylation and increase in H3K9 dimethylation in fetal and neonatal life [55]. Ultimately with accumulation of H3K9 dimethyl marks, DNA methyltransferase 3A (DNMT3A) is recruited to the promoter, which initiates *de novo* DNA methylation leading to permanent reduction of *Pdx1* gene expression in adulthood [67]. Similarly, increased DNA methylation of key enhancer element and marked reduction in *Pdx1* gene expression have also been reported in islets of humans with T2D [70]. These findings indicate that epigenetic modifications at this locus in islets contribute significantly to diabetic phenotype both in humans and animals. As in islets, UPI appears to reduce genome-wide DNA methylation and increase histone H3 acetylation in UPI offspring liver [71]. Additionally, site-specific increased histone H3 acetylation and reduced nuclear protein levels of HDAC1 and HDAC activity have been identified in promoter sequences of peroxisome proliferator-activated receptor-γ (PPARγ) coactivator (*Pgc1*) and carnitine palmitoyl transferase I (*Cpt1*) in the same rats [72] and correlated with postnatal gene expression levels and subsequent development of insulin resistance in the UPI offspring [73]. However, despite finding genome-wide and loci-specific changes in epigenetic marks in UPI tissues, it remains to be determined whether these changes are causative for the abnormal metabolic phenotype observed in this model with late onset of T2D. Importantly, in the UPI model, the epigenetic process was found reversible at the neonatal stage. Simmons and colleagues first found that administration of Exendin-4 (Ex-4; a long-acting GLP-1 analog currently being used to treat humans with T2D) during the postnatal period, prevented the development of late diabetes in UPI offspring. This was correlated with restoration of *Pdx1* expression and a return of the beta cell mass to normal level [74]. Importantly they found that Ex-4 was able to reverse epigenetic modifications in the UPI offspring. Ex-4 interrupted the self-propagating aberrant epigenetic cycle in UPI islets by restoring histone acetylation and H3K4 methylation, thereby preventing H3K9 and DNA methylation and finally normalizing *Pdx1* expression. This study elegantly shows that an acute pharmacological intervention during a critical window may be sufficient to reverse aberrant epigenomic marks and prevent metabolic impairment in offspring.

## 4. How to Explain Risk Inheritance via the Maternal Line?

Numerous animal studies have confirmed the detrimental effects of F0 maternal overnutrition/undernutrition exposure on F1 offspring’s glucose metabolism in adulthood. There are now also indications that glucose metabolism is altered in the F2 offspring as well as grand-offspring (F3) of in utero malnourished F1 females, even when the F1 and F2 females were well-nourished after weaning [47,75]. While aiming to identify the relative parental contributions that lead to F2 offspring outcomes in models of maternal malnutrition, it was reported that F1 males exhibit moderate hyperglycemia as well as IGT with ageing and impaired glucose-stimulated insulin secretion, and that all F2 offspring of either F1 males or females develop IGT [76]. This provides the experimental proof that inter-generational transmission of IGT may also arise through paternal lineage and not exclusively via the more widely accepted maternal and grand-maternal inheritance [6,75,76].

**Figure 2:**
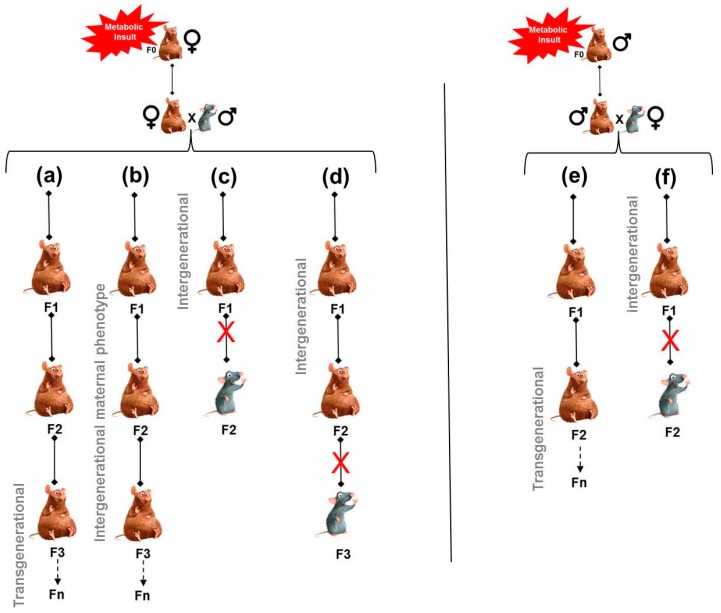
Non-genetic transmission of parental programming effects. Left panel: Maternal exposure to a metabolic insult (such as an unbalanced diet, obesity, diabetes) (brown rodent) influences both her developing fetus in utero (F1) as well as the developing germ cells in the fetus which later give rise to F2, both of which are present at the time of environmental exposure. When phenotypes do not persist after F1 or F2, such a programming effect is referred to as intergenerational inheritance (c,d). However, the cells which form the F3 generation are not present at the time of environmental exposure. If they still carry phenotypes in the F3 generation, they do so by transgenerational inheritance (a). This transgenerational inheritance (non-genetic) appears to be mediated through the transmission of epigenetic information through the germline [6,80,81]. Another mechanism for the transmission of programming effects is that programmed changes in the F1 mother (maternal physiology and behavior) influence the F2 offspring development, and so on. In this case, the metabolic insult is the altered maternal phenotype and the altered phenotype is established de novo in each new generation (b). Such a programming effect is referred to as maternal phenotype intergenerational inheritance (b). Right panel: For paternal exposure to a metabolic insult (brown rodent), only the sperm cells which form the F1 generation are present and directly exposed to the insult. When phenotypes do not persist after F1, the programming effect is referred to as intergenerational inheritance (f). When phenotypes persist into F2 and beyond, it is referred to as transgenerational inheritance (e). This transgenerational inheritance (non-genetic) appears to be mediated through the transmission of epigenetic information through the paternal sperm cells [6,80,81].

As a matter of fact, the intergenerational inheritance of disease risk may be mediated by various non-genomic mechanisms such as (1) persistence of maternal exposure to the external stressor, (2) indirect mechanisms associated with parental physiology [77], and (3) epigenetic mechanisms [6,78,79,80] (Figure 2).

### 4.1. Persistence of Maternal Exposure to Adverse Environmental Conditions along Generations

In some cases, developmentally programmed traits may simply be the result of persistent or replicated exposure during critical periods of development, generation after generation. It has been suggested that the history of severe socio-political disruptions and economic disadvantage suffered by minority populations, represents such recurring, multigenerational environmental insults (including chronic food insecurity) that may have led to developmentally programmed traits that contribute to the cardiometabolic alterations [82]. Comparable generational effects have also been demonstrated in experimental animal studies in which environmental stressors (inappropriate diet, lack of exercise, endocrine disruptor) are reintroduced to offspring over successive generations.

### 4.2. Persistence of an Altered Maternal Phenotype along Generations

Another potential mechanism for the transmission of programming effects is where programmed changes in the mother influence the development of her offspring during pregnancy. Thus, the phenotype is established *de novo* in each new generation [47]. Maternal undernutrition during pregnancy (F0) increases the risk of diabetes and obesity in the F1 offspring, and when these high-risk adult F1 females are themselves pregnant, the metabolic stress of pregnancy may result in hyperglycemia and/or overt gestational diabetes that, in turn, may contribute to a defective beta cell mass and increased diabetes risk in F2 offspring [47]. By this mechanism, primary maternal malnutrition (F0) may be converted into F1 gestational diabetes, which may then be passed on from this (F1) generation to the next (F2, F3 and so on). In this scenario, the intergenerational transmission of phenotypes would arise exclusively through the maternal lineage. Such a mechanism may contribute to the diabetes propagation in the GK rat model of spontaneous T2D: it offers a rationale for the initiation of pancreatic programming in the F1 offspring of the founders (F0) and its subsequent transmission across generations, as the GK line arose from intercrosses between Wistar females and males with borderline IGT, but otherwise normal basal blood glucose levels [51].

### 4.3. Involvement of Non-Genomic (Epigenomic) Modifications Induced by the Maternal Phenotype and Transmitted via the Oocyte

There is now experimental evidence that maternally-induced non-genomic alterations can be transmitted through generations, and represents a vicious cycle that could lead to future generations becoming obese or diabetic without any exposure to suboptimal early nutrition.

It has been reported that the peroxisome proliferator-activated receptor alpha and glucocorticoid receptor promoters are hypomethylated in the liver of protein-restricted female rats in both F1 and F2 generations issued from protein-restricted F0 female rats [83]. To be considered a true transgenerational phenotype transmitted by epigenetic modification of the germline, the phenotype must be present in the F3 generation, which is the first non-exposed generation [84]. For intergenerational inheritance to take place, these induced epigenetic modifications must survive further epigenetic reprogramming both in the germline and following fertilization. There is some evidence that maternal malnutrition can adversely affect glucose/insulin metabolism in the F3 generation [85]. These findings imply that altered epigenome modifications induced by altered maternal metabolism, belong to the oocyte and are propagated via the germ line.

To directly show that the maternally-triggered defects in the oocyte program metabolic disease in the offspring, Huypens et al. [86] used a model of obese and glucose intolerant female mice (HF diet). To determine whether these metabolic traits were inherited by the offspring through germline effects, oocytes from obese females were fertilized with sperm from lean males, and transferred at the two-cell stage into the oviducts of surrogate females fed a normal diet. This approach eliminates any physiological effects that maternal obesity may have had on the embryo during gestation, as well as the possible postnatal factors such as lactation and maternal behavioral effects associated with an obese lactating mother. Therefore, any metabolic phenotypes observed in the resulting offspring would solely be attributed to defects in the oocytes. Doing so, Huypens et al. observed that female as well as male offspring derived from obesity-exposed oocytes were more likely to become obese than control offspring. Both female and male offspring were glucose intolerant and insulin resistant. Overall, this study demonstrates that the metabolic effect in offspring can be attributed to factors within the oocyte that are independent from the mother’s uterine environment.

It is well recognized that maternal obesity or diabetes can have a profound impact on the female fertility as evidenced by alterations of hormone levels, anovulation, and oocyte quality [87]. Obese or diabetic female mice have increased rates of ovarian apoptosis, less mature oocytes after ovulation, slower preimplantation embryo development, and perturbed embryo differentiation in inner cell mass and trophectoderm lineage [88,89]. Maternal diet-induced obesity also alters oocyte and embryo mitochondrial metabolism as evidenced by altered mitochondrial distribution, increased mitochondrial membrane potential and mitochondrial DNA content, coupled with increased reactive oxygen species and depleted glutathione (indicative of oxidative stress) [90], and altered mitochondrial ultrastructure [91]. Increased lipid content associated with cellular oxidative stress has been reported in oocytes from obese mice [91,92]. A decrease in the expression of genes encoding two key metabolic receptors, the glucose transporter Glut-1 and the low-density lipoprotein receptor, has also been observed, which may be indicative of a mechanism that operates against excessive nutrient uptake by the blastocyst [93]. Collectively, these studies suggest that various lesions/marks acquired by the oocyte in response to the maternal metabolic status prior to fertilization, are sufficient to alter the offspring phenotypes.

Among these lesions, mitochondrial inheritance is a candidate to explain the effect of maternal obesity on offspring health, since exposure of oocytes to an obese environment decreases mitochondrial DNA content [94] and function [90]. These defects persist in the mitochondria of fetal livers and kidneys [94], thus potentially linking maternal diet to offspring’s metabolism via maternally-derived mitochondrial transmission.

Abnormal DNA methylation in oocytes [95] and poor placentation [57] are correlated to maternal obesity in mice. As placental development requires proper oocyte DNA methylation [96], one can also speculate that maternal obesity disrupts the epigenetic landscape in oocytes, leading to placental dysfunction and later-on to offspring metabolic disorders.

Finally, similar to what is known for sperm [97,98,99] (see [6]), maternal obesity or diabetes prior to conception may alter the expression of noncoding RNAs in the oocyte. These modified elements can escape epigenetic reprogramming at fertilization, therefore become heritable, and finally contribute to the alteration of the developmental trajectory of the embryo.

## 5. Obesity or Diabetes Risk Can Be Transmitted across Generations via the Paternal Line. Epidemiological and Experimental Evidence

Paternal inheritance of obesity/diabetes [100] was first suggested by epidemiological analysis of human cohorts and was recently named Paternal Origins of Health and Disease (POHaD) [101].

For instance, the offspring of fathers who had been undernourished during the 1944–1945 famine in The Netherlands developed increased adiposity more frequently than controls [102,103] and this up until the second generation. Furthermore, in the Overkalix cohort study [104], a northern Swedish community that endured year-to-year food supply variations, had increased diabetes frequencies which have been related to the grandfathers’ food availability.

**Figure 3:**
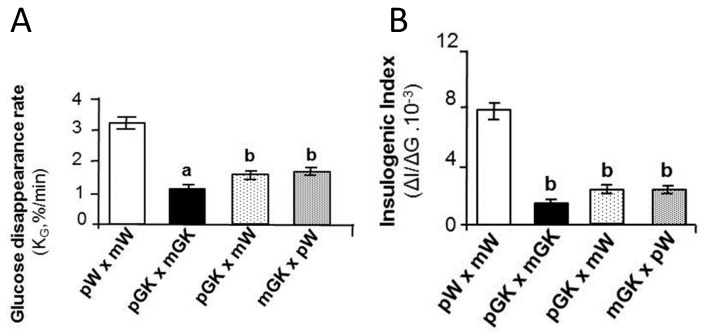
Metabolic and endocrine phenotypes of young male rats issued from crosses (F1) between normal Wistar (mW) or diabetic (mGK) mothers and normal (pW) or diabetic (pGK) fathers. Indices of glucose tolerance (rate of glucose disappearance, K_G_), after an in vivo glucose charge) (**A**), and of insulin secretion (insulinogenic index, ΔI/ΔG), in response to an in vivo glucose charge (**B**), have been evaluated. (a) *p* < 0.01 versus pW/mW, pGK/mW and mGK/pW. (b) *p* < 0.001 versus pW/mW and pGK/mGK. Adapted from [108]. The Goto-Kakizaki (GK) rat is one of the best characterized animal models of spontaneous type 2 diabetes (T2D) model. It has provided new insights into the pathogenesis of T2D as the rats develop spontaneous defects in insulin secretion and action and long-standing diabetes complications that, in many ways, resemble those described in human T2D [53,54].

The heritability of the obese/diabetic paternal phenotype was confirmed by experimental approaches. Multiple animal studies have now demonstrated that offspring’s metabolic phenotype is affected by paternal unbalanced diet. Female rats born to fathers on a HF diet had impaired pancreatic islet biology, insulin secretion and glucose tolerance in adulthood [105]. The F1 offspring of male mice fed a HF diet exhibited the same obese phenotype as their fathers [99,106]. The offspring metabolic phenotype can also be affected by paternal undernutrition. Male and female born to fathers fed a low protein and high sugar diet had increased hepatic expression of lipid biosynthetic genes [98]. Offspring metabolic phenotype can also be affected by paternal diabetes. Paternal low-dose STZ-induced diabetes in mice was accompanied by insulitis and insulin secretion deficiency in their F1 offspring [107]. Paternal T2D alone (i.e., without associated obesity) impairs early development of endocrine pancreas and adult tolerance du glucose in rat F1 offspring. This was previously suggested by our group using a spontaneous model of paternal T2D [46,108] (Figure 3).

To our knowledge, the most comprehensive study to evaluate the transgenerational effects of paternal diabetes on offspring and the mechanisms that mediate these effects, has been provided by Wei et al. [109]. Using a non-genetic diabetes mouse model (low dose of STZ combined to HF diet), this group showed that paternal diabetes did not alter body weight, fat mass, or energy intake in F1 offspring, but it induced fasting hyperglycemia, glucose intolerance and insulin insensitivity in the male offspring to an extent similar to that seen in their fathers. To determine the mechanisms of the glucose intolerance and insulin insensitivity observed in the F1 male offspring, Wei et al. performed genome-wide microarray analyses of their pancreatic islets. The expression of 402 genes was modified (97 up-regulated and 305 downregulated). A large proportion of these genes were related to insulin and glucose metabolism, including GTPase activity, GTP and ATP binding, sugar binding, and calcium binding. Wei et al. also found several differentially methylated loci in the F1 islets. The same group also asked whether the metabolic and epigenetic changes in the F1 generation can be passed to the next generation (F2 generation). For that purpose, they mated F1 diabetic males (F1-D) whose fathers were diabetic, with normal females, and then examined metabolic and epigenetic changes in their offspring (F2). The F2 generation also exhibited impaired glucose tolerance and decreased insulin sensitivity (but not fasting hyperglycemia). Examination of the methylation status for 10 regions distributed on different chromosomes that were most affected by paternal diabetes, showed that all of these regions were still significantly affected in the F2 generation. As the F1 animals received normal diet without any STZ treatment and their F2 offspring exhibited similar phenotypic and epigenetic changes, the observed effects of epigenetic inheritance are most likely attributable to the diabetes-associated physiological and metabolic conditions in F0 male founders.

## 6. Paternal Obesity or Diabetes Remodels Sperm Epigenome

### 6.1. Remodeling of DNA Methylation Profile

To identify potential sperm DNA methylation marks associated with transgenerational effects, De Castro Barbosa et al. [110] have compared the DNA methylation profile of the sperm cells from F0 mice fed a HF diet and their F1 offspring fed a normal diet (C). They found 18 loci differentially methylated in F0-HF sperm and F1-C sperm in comparison with their respective controls. The next important question they addressed in the same study, is whether paternal metabolic state-induced methylation changes in sperm-inherited genes can be inherited to other somatic tissues. To that aim, they compared the DNA methylation profiles in F2 offspring adipose tissue in regions corresponding to the differentially methylated regions altered in sperm. They concluded that none of these regions showed differential methylation, despite altered gene expression in adipose tissue. This suggests that differential methylation of genes inherited from sperm, does not concern somatic cells of offspring, or alternatively, that it is not maintained in the somatic cells. Others also concluded that the link between differential methylation of parental sperm and the altered methylation and gene expression in somatic tissues of adult offspring, was weak, if any [98,111].

Does paternal diabetes similarly alter the sperm methylome? A positive answer is brought about by the Wei et al. study [109]. They observed that a large proportion of differentially methylated genes identified in F0-D sperm overlapped with that in offspring pancreatic islets. They concluded that cytosine methylation in sperm (especially for intragenic regions) is a strong factor in determining methylation status in somatic tissues. As an example, Wei et al. found that the same region of the *Pik3ca* gene was hypermethylated in F0-D sperm as well as in F1-D islets (*Pik3ca* gene codes for the p110α protein which is the catalytic subunit of the kinase PI3K). To know whether genes differentially methylated in the offspring are de novo methylated in the embryo or potentially inherited from sperm, Wei et al. analyzed the methylome in E3.5 blastocysts: *Pik3ca* showed higher methylation levels in F1-D blastocysts compared with F1-Control (F1-C) blastocysts, suggesting that this gene partially inherits methylated alleles from sperm. To sum-up, Wei’s results suggest that cytosine methylation status in diabetic sperm, strongly predisposes toward methylation in blastocysts, probably due to incomplete post-fertilization demethylation. Finally, Wei et al. found that several Differentially Methylated Regions (DMRs) distributed on different chromosomes in F1-D islets, were still significantly affected in F2-D islets, and that the methylation status of Pik3ca was also similar to that found in the F1 generation. This suggests that in this model, the epigenetic changes established in the F1 generation can be passed to the next generation (F2), despite the well-recognized genome-wide demethylation of the parental genomes during preimplantation embryo development. However, while these studies showed that altered DNA methylation signature of spermatozoa from HF male could be passed through the progenies, a recent study strongly indicated that sperm methylome was shaped by genetic and epigenetic variation but not by diet, thus disproving the idea that DNA methylation is the vector of epigenetic inheritance in this experimental HF model [112].

While many experimental studies focus on the epigenetic modifications involved in the inheritance of the newly acquired metabolic pathologies, few of them have been performed in humans. A recent study performed an epigenome profiling of sperm from lean and obese men [113]. While similar histone positioning was found, sncRNAs (tRFs and piRNAs) expression and DNA methylation patterns were markedly different between lean and obese men. In a separate cohort of morbidly obese men, gastric-bypass-induced weight loss was associated with a dramatic remodeling of sperm DNA methylation, notably at genetic locations implicated in the central control of appetite [113]. These data provide evidence that is similar to what happens in rodent models, the epigenome of human sperm cells dynamically changes under environmental pressure. However, to what extent, if any, these gametic epigenetic changes in obese men influence the metabolic profile of their offspring remains unknown.

### 6.2. Remodeling of Chromatin

Sperm DNA is mainly associated with highly basic proteins called protamines (protamine 1 and 2). The replacement of histone by protamine permits the typically condensed nuclear structure in mature spermatozoa, where DNA becomes organized in compacted units, similar to nucleosomes [114]. At the functional level, this highly compacted chromatin structure induces a sperm genome-wide transcriptional repression. A small amount of histones (1% to 10%) is thought to remain in contact with DNA and to shuttle to the next generation of offspring. Several studies suggest that paternal dietary exposure can modulate histone composition at regulatory genes implicated in developmental processes. Using a model of paternal HF diet in mice, Terashima et al. [115] confirmed that alteration of hepatic mRNA level of seven genes in the offspring depended on paternal diet. Using chromatin immunoprecipitation followed by high-throughput sequencing, they reported that this pattern was associated with differential histone H3-retention at genes involved in the regulation of embryogenesis and differential H3K4me1-enrichment at transcription regulatory genes in sperm of HF fathers vs. controls. In spermatids from mice with HF diet-induced obesity, a decreased level of SIRT6 has been reported [116]. SIRT6 is a protein with ADP ribosyltransferase and histone H3 deacetylation (HDAT) activities and it is expressed in the elongated spermatids. This decreased SIRT6 expression was associated with an increased level of acetylated H3K9 and increased DNA damage in the corresponding spermatid nucleus [115]. The fate of paternal histones and histone modifications after fertilization remains unknown, but some retained histones stay associated with the paternal genome in the zygote. Öst et al. [117] demonstrated that paternal diet intervention modifies offspring’s chromatin state in drosophila which require H3K9/K27me3-dependent reprograming of metabolic genes in sperm and zygote, indicating that histones and their modifications could resist further reprograming and directly influence gene expression in differentiated cells and be propagated into the subsequent generations.

### 6.3. Remodeling of the Small Non-Coding RNA Expression Pattern

There is recent compelling evidence that small noncoding RNAs (sncRNAs) play a crucial role in the intergenerational inheritance of the metabolic phenotype. sncRNAs can regulate DNA methylation, histone modifications, and mRNA transcription [118] and can induce non-Mendelian transgenerational inheritance in mammals [119,120,121,122]. Mouse, rat and human spermatozoa [123,124] contain an important variety of small RNA subtypes, notably ribosomal RNA (rRNA), microRNAs (miRNA), PIWI-interacting RNAs (piRNA), small nucleolar RNA (snoRNA), small nuclear RNA (snRNA) and tRNA-derived fragments (tRFs). tRFs derive from 5′ tRNA halves and are 30 to 34 nucleotides in size.

Several studies have reported alterations of tRFs, piRNA or miRNAs expression profile in sperm from obese or diabetic rodents [106,124,125,126].

## 7. Mechanisms for the Transmission of Risk via the Paternal Line

### 7.1. Transferred by Sperm. Proof of Concept

To assess the direct contribution of sperm cells in the transmission of obesity/diabetes to the next generation, the relevant method is to perform in vitro fertilization (IVF) of normal oocytes by sperm cells from obese or diabetic males, and to transfer the oocytes into surrogate mothers [86,97]. Using such a strategy to ensure exclusive inheritance via the gametes, Huypens et al. [86] showed that the progenies (maintained on normal diet) derived from embryos fertilized in vitro by sperm from HF males developed obesity, glucose intolerance and insulin resistance in adulthood. Therefore, it was proposed that the F0-HF sperm itself contains sufficient information to transmit a set of acquired metabolic disorders such as glucose intolerance and insulin resistance to offspring.

Paternal prediabetes could potentially affect the offspring’s metabolism via a number of different mechanisms. For example, paternal lifestyle changes can affect spermatogenesis and the composition of seminal fluid [127,128]. Studies in humans and mice have demonstrated that increased paternal body mass index is associated with reduced sperm motility [129], increased incidences of sperm abnormality [130] and DNA fragmentation [131], and reduced pregnancy rates [132]. In rodents, HF [133] or high-energy [134] diet impacts negatively on sperm motility, sperm DNA integrity, and blastocyst development. In men, paternal obesity has been shown to associate with decreased blastocyst development [135]. The molecular mechanisms underlying the modality of transmission of paternal obesity/diabetes are obviously non-genetic, as the experimental studies were carried out in isogenic animals. The frequent occurrence and heritability of these metabolic disorders preclude Mendelian transmission of mutational events and are reminiscent of the epigenetic heredity documented in various organisms, ranging from *Caenorhabditis elegans* to humans [136]. Since paternal obesity or diabetes remodels the sperm epigenome, the hypothesis that the father’s environmental information to control the offspring’s phenotype is carried out by epigenetic heredity via paternal sperm has been successfully addressed in several recent experimental studies.

### 7.2. Sperm-Borne RNAs Are Candidates That Could Transfer the Risk of Obesity or Diabetes. Proof of Concept

To determine whether RNAs could be the vector of paternal traits inheritance, Grandjean et al. [99] performed microinjection of either sperm or testis RNAs from F0-HF (spermRNA-HF and testisRNA-HF respectively) or F0-control male mice, into normal mouse one-cell embryos. The males generated by testisRNA-HF and spermRNA-HF had increased body weight, altered glucose tolerance and decreased insulin sensitivity compared to those generated from microinjection of sperm or testis RNA from F0-control fathers. The testisRNA-HF males had fasting blood glucose levels that were significantly greater than the testisRNA-control males, a trait not found in the spermRNA-HF.

### 7.3. Transfer of Paternal Metabolic Traits by Sperm tRNAs

Two independent studies recently evaluated the potential contribution of sperm cleaved tRNA (tRFs) from obese male rodents to metabolic alterations of their offspring. Full-length tRNAs are associated with ribosomes during the synthesis of new proteins, but tRFs previously described in sperm [137] still exert elusive function. In both studies, analysis of control sperm revealed an abundance of 50 tRFs, ranging from 64% to 80% of all small RNAs [97,138]. Since an enrichment in tRFs profile was found in sperm from obese male mice, Chen et al. [97] injected an enriched (but not pure) tRFs preparation from sperm of HF males into normal zygotes. The tRF-HF offspring exhibited impaired glucose tolerance, but no insulin resistance. This suggests that paternal diet may affect glucose metabolism in the next generation via sperm tRFs, although another epigenetic mechanism is required to fully establish the phenotype. To what extent are sperm tRFs from obese mice able to alter early offspring development? To answer this question, Chen et al. [97] have evaluated the transcriptional profiles of pre-implantation embryos receiving microinjected sperm tRFs. They showed that hundreds of genes were up- or down-regulated in eight-cell and/or blastocyst-stage embryos derived from zygotes injected with sperm tRFs from males fed a HF diet. The down-regulated genes were specifically enriched for metabolic regulatory pathways [97]. A transcriptomic analysis of pancreatic islets in tRF-HF adults uncovered downregulation of genes involved in ketone and carbohydrate metabolism [97]. Finally, Chen et al. [97] identified RNA modifications (5-methylcytodine and N2-methylguanosine) that were more prevalent in tRFs isolated from sperm of HF males than from those of control males. When synthesized without these modifications, these tRFs were unable to induce metabolic disorders, likely due to reduced RNA stability. It is postulated that tRFs generated in sperm of HF males, might resist degradation and affect metabolic gene expression through embryo to adulthood via a transcriptional cascade effect. Overall, these experimental studies provide evidence for tRFs as epigenomic messengers in the epigenetic transmission of information from father to offspring.

The occurrence of tRFs in human sperm is yet to be determined, though they are present and functional in other human cell types [139].

### 7.4. Transfer of Paternal Metabolic Traits by Sperm miRNAs

Sperm-borne miRNAs are important for embryo development, as evidenced by embryonic arrest in embryos deficient in sperm-derived miRNA [140]. Moreover, single miRNAs injected directly into fertilized one-cell embryo induce phenotypes in adult offspring, such as cardiac hypertrophy for miRNA-1 [141], coat color change for miRNA-221/222 [120], embryo and offspring overgrowth for miRNA-124 [119].

In the RNA analysis of sperm from F0-HF mice, Grandjean et al. [99] identified miR-19b and miR-29a as the two most abundant deregulated miRNAs. These two candidates were injected separately into fertilized one-cell embryos to test for their potential as obesity inducers. While body weight of miR29a adults was normal, all the miR19b adults (either males or females) were obese. Despite overweight, all the miR19b mice showed normal fasting glucose levels (no overt diabetes). However, half of the obese miR19b animals had impaired glucose tolerance and insulin sensitivity. Analysis of the progenies (designated F1-miR19b) of individual miR19b males crossed with control females showed that the F1-miR19b mice developed the full miR19 phenotype (obesity and fasting hyperglycemia) despite the normal metabolic features of their miR19b progenitors. These results suggest that upon RNA injection, epigenetic changes take place which can remain phenotypically silent (miR19b normal males), but are later transmitted to the progeny (F1-miR19b obese males). One must admit that the micro-injected RNA procedure does not truly mirror the level of a given RNA present in HF sperm, and it seems unlikely that a unique RNA is responsible for the whole phenotype. The amount of data so-far available rather favors the possibility that multiple sncRNAs play a role in the inheritance of diet-induced metabolic phenotypes, with the best candidates belonging presently to the tRFs and miRNA subtypes.

## 8. What Happens If Both Parents Are Obese or Diabetic?

The combination of having both parents obese or diabetic on fecundity and offspring health, has received minimal attention. This information is not trivial, as the percentage of couples of reproductive age with both partners overweight/obese is increasing in most countries. To date, there have been few studies that have assessed the combined effects of maternal and paternal obesity on pregnancy and fetal health. Two studies in human assisted reproductive technology cohorts found no effect of maternal and paternal obesity on pregnancy establishment [142,143]. However, in a rodent model of obesity (HF diet), insulin resistance and liver steatosis were greatest in offspring where both parents had been fed a high-fat diet prior to and during gestation compared with just one parent [144].

Moreover, parental obesity in mice (HF diet) reduces fetal and placental weights without altering pregnancy establishment and was not dependent on an *in utero* exposure to maternal obesity. This may result from perturbed early embryo and fetal health, since reduced embryo developmental competency, reduced blastocyst cell numbers, impaired mitochondrial function, and alterations to active and repressive embryonic chromatin marks were found and were associated to aberrant placental gene expression and reduced fetal liver mtDNA copy numbers [145]. These findings show that the effects seen in offspring phenotypes as a result of combined paternal and maternal obesity are additive (and not potentiator) and are likely due to the accumulation of both paternal and maternal effects on embryo and fetal development. Using the GK rat model of T2D, our group came to a similar conclusion concerning the combined effect of maternal and paternal T2D on the programming of the offspring glucose tolerance and insulin secretion [108] (Figure 3).

## 9. Conclusions and Questions

It is clear that both the paternal and the maternal lineages are responsible for more than just their genetically encoded information. Several recent experimental studies have convincingly shown that obesity or diabetes in the father induces epigenetic alterations in sperm that can be inherited for several generations, and finally alters epigenetic marks in offspring somatic tissues. These epigenetic factors which are heritable, should be regarded as important as genetic factors in determining the risk for obesity and diabetes. Similarly, the passage of epigenetic information through the oocytes and originating from the obese or diabetic mother, is attractive and can explain the transmission of programmed effects to at least a second generation through the female lineage. However, it is unclear how often this might result in the induction of true oocyte epigenetic effects that are transmissible across several generations. Furthermore, one must not to forget alternative mechanisms for the intergenerational inheritance of the programmed phenotype via the maternal line, including maternal exposure to persisting environmental factors and maternal effects such as altered maternal physiology. Besides the inherited traits from parental obesity/diabetes extensively discussed in this review, epigenome modifications can also arise from a variety of environmental exposures including undernutrition, stress, physical activity and toxins, and they could separately or additively also contribute to the inheritance of the obese/diabetic phenotypes [78,79].

While our understanding of epigenetic contribution to the development of obesity and diabetes expands rapidly, there is much work to do to resolve the entire process. Are epigenetic germ cell modifications the result of direct effects on the germ cells or of effects induced in the soma being transmitted to the germ cells? How are such modifications protected from epigenetic reprogramming? Are DNA methylation, histone codes or ncRNAs involved? What makes one locus epigenetically stable and another environmentally susceptible? Do different mechanisms operate through oocytes and sperm cells? Do epigenetic germ cell modifications influence early preimplantation embryonic development, later stage embryonic growth, tissue-specific stem cells? What are the critical developmental periods? Are there differences depending on the tissue? Can interventions be targeted to specific epigenetic markers? Finally, the many limitations of the existing epigenetic studies do not allow to answer the crucial question whether epigenetics is a cause, a consequence or a correlation. Today, the relative importance of any or all of these issues in human populations is unknown. Thus, elucidation of these mechanisms and validation of interventions that improve health in men or women of reproductive age and potentially interrupt vicious cycles of disease risk transmission to their descendants, are critical public health and research issues. They bear the potential to halt the diabetes epidemic encountered throughout the world at present.
